# Lack of physiological evidence for cytochrome filaments functioning as conduits for extracellular electron transfer

**DOI:** 10.1128/mbio.00690-24

**Published:** 2024-04-02

**Authors:** Ingrid A. Schwarz, Baha Alsaqri, Yassir Lekbach, Kathryn Henry, Sydney Gorman, Trevor Woodard, Laura Dion, Lauren Real, Dawn E. Holmes, Jessica A. Smith, Derek R. Lovley

**Affiliations:** 1 Department of Biomolecular Sciences, Central Connecticut State University, New Britain, Connecticut, USA; 2 Department of Microbiology, University of Massachusetts Amherst, Amherst, Massachusetts, USA; 3 Department of Physical and Biological Sciences, Western New England University, Springfield, Massachusetts, USA; University of Delaware, Newark, Delaware, USA

**Keywords:** electromicrobiology, geomicrobiology, extracellular electron transfer, microbial nanowires, *Geobacter*

## Abstract

Extracellular cytochrome filaments are proposed to serve as conduits for long-range extracellular electron transfer. The primary functional physiological evidence has been the reported inhibition of *Geobacter sulfurreducens* Fe(III) oxide reduction when the gene for the filament-forming cytochrome OmcS is deleted. Here we report that the OmcS-deficient strain from that original report reduces Fe(III) oxide as well as the wild-type, as does a triple mutant in which the genes for the other known filament-forming cytochromes were also deleted. The triple cytochrome mutant displayed filaments with the same 3 nm diameter morphology and conductance as those produced by *Escherichia coli* heterologously expressing the *G. sulfurreducens* PilA pilin gene. Fe(III) oxide reduction was inhibited when the pilin gene in cytochrome-deficient mutants was modified to yield poorly conductive 3 nm diameter filaments. The results are consistent with the concept that 3 nm diameter electrically conductive pili (e-pili) are required for *G. sulfurreducens* long-range extracellular electron transfer. In contrast, rigorous physiological functional evidence is lacking for cytochrome filaments serving as conduits for long-range electron transport.

**IMPORTANCE:**

Unraveling microbial extracellular electron transfer mechanisms has profound implications for environmental processes and advancing biological applications. This study on *Geobacter sulfurreducens* challenges prevailing beliefs on cytochrome filaments as crucial components thought to facilitate long-range electron transport. The discovery of an OmcS-deficient strain’s unexpected effectiveness in Fe(III) oxide reduction prompted a reevaluation of the key conduits for extracellular electron transfer. By exploring the impact of genetic modifications on *G. sulfurreducens*’ performance, this research sheds light on the importance of 3-nm diameter electrically conductive pili in Fe(III) oxide reduction. Reassessing these mechanisms is essential for uncovering the true drivers of extracellular electron transfer in microbial systems, offering insights that could revolutionize applications across diverse fields.

## INTRODUCTION

With few exceptions (1), discoveries of *Geobacter sulfurreducens* extracellular cytochrome-based filaments have concluded that these cytochrome nanowires are the most important conduits for this microbe’s remarkable long-range extracellular electron transfer capabilities (2–6). This conclusion is in accordance with the well-known role of cytochromes in electron transport and the finding that some purified cytochrome filaments are electrically conductive. However, conductance in response to applied external voltages is not rigorous evidence that cytochrome filaments are involved in the physiological process of long-range electron transport. A more direct functional analysis of a role in extracellular electron transport is required.

*G. sulfurreducens* long-range extracellular electron transfer is best evaluated with analysis of Fe(III) oxide reduction. This is because *G. sulfurreducens* requires electrical connections with an extracellular volume 20–50 times the size of the cell in order to access sufficient Fe(III) oxide to gain enough energy for replication (7). Only electrical connections achieved with nanowires are energetically feasible (7). This assessment is in accordance with the observation that Fe(III) oxides primarily bind to nanowire networks rather than the cell surface during growth on Fe(III) oxides (8). *G. sulfurreducens* current production on electrodes may involve some long-range electron transfer, but the most metabolically active cells in electrode biofilms are near the surface (9). Thus, it is inappropriate to infer the extent of long-range electron transfer from current production levels. Biofilm conductivity, which measures electron flux through the biofilm (10, 11), is a better indicator of the potential for long-range electron transport than current production. Thus, biofilm conductivity can provide insights to complement the functional interpretation of Fe(III) oxide reduction mechanisms.

Filaments of the *G. sulfurreducens c*-type cytochromes OmcE, OmcZ, and OmcS have been detected in purified preparations of outer-surface proteins (1–5). OmcE filaments have not been recovered from wild-type cells, suggesting that they are a mutant artifact. Significantly, the surface of OmcE is glycosylated, which is expected to insulate the filaments from electrical contact with extracellular electron acceptors (3, 6). No conductivity measurements have been made to demonstrate electron transport along the length of OmcE filaments (3). Furthermore, functional genetic studies have demonstrated that OmcE is not required for Fe(III) oxide reduction (12). Deleting the gene for OmcE also had no impact on current production (13) and was associated with an increase in biofilm conductivity (11). These results are the opposite of the results expected if OmcE filaments were involved in long-range electron transport. Thus, there is currently no functional data supporting a role for OmcE filaments in long-range extracellular electron transfer.

Functional data also does not support a role for OmcZ in long-range electron transfer. OmcZ is not required for Fe(III) oxide reduction (13). Deleting the gene for OmcZ did decrease current production (13), but this result cannot be attributed to an impact on long-range electron transport through the biofilms. OmcZ is not dispersed throughout current-producing biofilms, as would be required for long-range electron transport, but rather is highly concentrated at the biofilm/electrode interface (14). Furthermore, the biofilms of multiple *G. sulfurreducens* strains that produced higher biofilm conductivities than the wild-type strain contained less OmcZ than the wild-type (11). These results are the opposite of what would be expected if OmcZ was the conduit for long-range electron transfer through biofilms.

OmcS filaments account for 10% of the filaments emanating from wild-type cells (15). OmcS filaments are conductive (4, 15). However, the suggestion that OmcS filaments are conduits for electron transport through current-producing biofilms (4) is inconsistent with the findings that (i) an OmcS-deficient mutant produced current as well as the wild-type strain (13) and (ii) deleting the OmcS-gene-actually-increased biofilm conductivity (11). Deleting the gene for OmcS was reported to prevent the reduction of Fe(III) oxide in *G. sulfurreducens* strain DL-1 (12). However, the essential requirement for OmcS in extracellular electron transfer has been brought into question with more recent findings that deleting the gene for OmcS resulted in only temporary or partial inhibition of Fe(III) oxide reduction in other strains of *G. sulfurreducens* (16, 17). These findings indicate that there are other routes for long-range electron transport.

An alternative hypothesis for *G. sulfurreducens* long-range electron transport is electron transfer along 3 nm diameter electrically conductive pili comprised of the pilin monomer PilA (8, 18–21). Multiple lines of evidence support PilA assembly into conductive 3 nm diameter filaments. An *Escherichia coli* strain expressing the *G. sulfurreducens* PilA gene produced 3 nm diameter electrically conductive filaments (22). The PilA gene could be precision-engineered for specific sensing functions by altering the amino acid content encoded in the PilA gene (23). Heterologous expression of the *G. sulfurreducens* PilA gene in *Pseudomonas aeruginosa* (24) and *Shewanella oneidensis* (25) also yielded 3 nm diameter conductive filaments whose properties could be changed by altering the PilA gene sequence. Modifying the pilin gene of *Cupriavidus necator*, an aerobe without outer-surface *c*-type cytochromes, to mimic the *G. sulfurreducens* PilA yielded a strain that produced conductive pili (26).

Filaments with the same 3 nm diameter and conductance as those heterologously expressed in *E. coli* (22) account for 90% of the filaments emanating from wild-type *G. sulfurreducens* (15). Modifying the PilA gene to encode carboxyl end peptide tags yielded strains of *G. sulfurreducens* that displayed filaments with those tags (27). This result demonstrates that the filaments are not extracellular DNA or cytochrome filaments. The conductivity along the length of *G. sulfurreducens* 3 nm diameter filaments has been tuned over a million-fold (40 µS/cm to 277 S/cm) simply by modifying the aromatic amino acid content encoded in the *G. sulfurreducens* PilA gene (28–30). Expressing a pilin gene encoding low aromatic amino acid abundance yielded a strain with low-conductance 3 nm diameter filaments emanating from the cells but with no change in the expression or conductance of the OmcS filaments (15). It is implausible that the wide range of conductivities associated with pilin modifications could be attributed to the expression of extracellular DNA or cytochrome filaments with different conductivities that tracked in unison with the changes in the aromatic amino acid content of the different pilins in multiple strains (31). Thus, here we conform with previous convention and refer to the 3 nm diameter conductive filaments as electrically conductive pili (e-pili).

It has been suggested that 3 nm diameter e-pili cannot exist because they have not been observed in cryo-electron microscopy preparations (2–6, 32, 33), but this appears to be due to a procedural limitation that fails to capture the 3 nm diameter filaments. The recovery of 6 nm diameter filaments comprised of PilA and another protein (3, 32) is an artifact of the mutant strains in which they were observed because they have never been observed emanating from wild-type cells. PilA, but not the protein that combines with PilA to form the 6 nm diameter filaments in mutant strains, is recovered from wild-type filament preparations (4, 29, 34, 35). This includes findings published by researchers who have subsequently claimed that wild-type cells do not express PilA-based filaments (4, 29, 35).

The functional evidence for a role of e-pili in extracellular electron transfer is that *G. sulfurreducens* strains that express PilA pilin genes modified to reduce nanowire conductivity do not reduce Fe(III) oxides or generate high current densities, even though outer surface cytochromes are properly localized (15, 16, 20, 21, 36). Furthermore, *G. sulfurreducens* biofilm conductivity is directly related to the abundance of PilA pilin, but not any of the cytochromes known to form filaments (11).

In the study reported here, we reexamined the OmcS-deficient strain that served as the basis for the original report that OmcS is required for Fe(III) oxide reduction (12). We also constructed new strains in which genes for multiple filament-forming cytochromes were deleted. The results indicate that e-pili, but not cytochrome filaments, are essential conduits for *G. sulfurreducens* long-range electron transport.

## RESULTS

### Effective Fe(III) oxide reduction in the absence of cytochrome filaments

**Fig 1:**
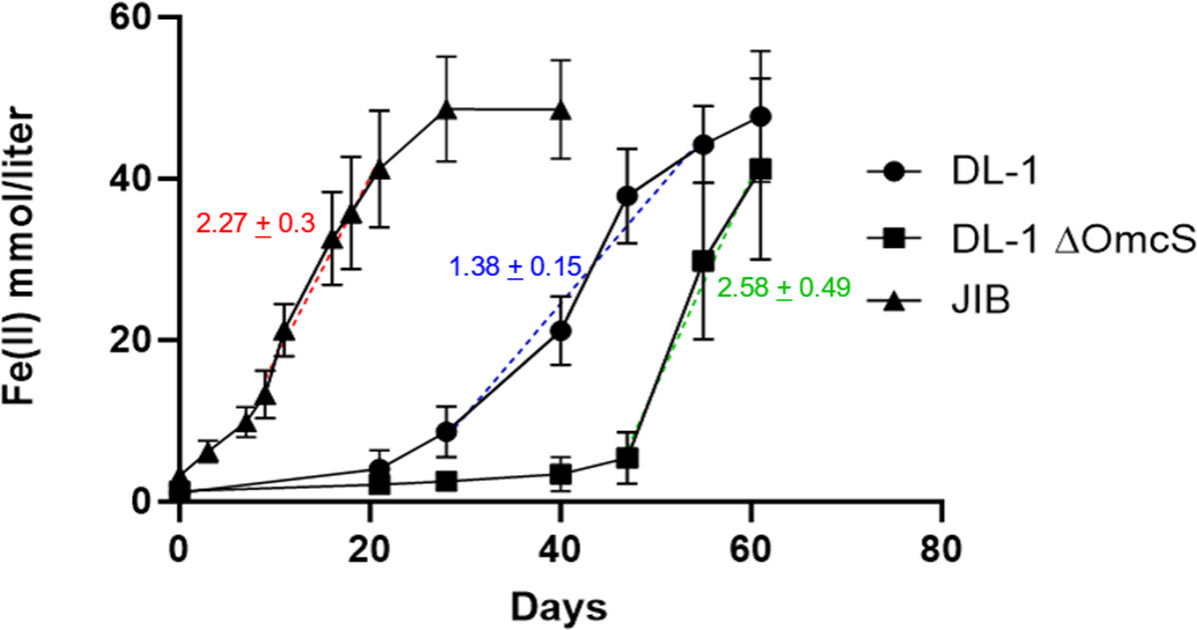
Fe(II) production from Fe(III) oxide reduction over time by the wild-type strain DL-1, the original DL-1/∆OmcS strain, and strain JIB. The slopes of the dashed lines are estimates of the fastest rates of Fe(III) reduction for each strain. Results are the means and standard deviations of triplicate cultures.

In a repeat of previously described studies (12), inocula of *G. sulfurreducens* strains grown with fumarate as the electron acceptor were introduced into medium with Fe(III) oxide as the electron acceptor (Fig. 1). The previously described DL-1/∆OmcS strain (12) was delayed in the initiation of Fe(III) oxide reduction compared to the parental DL-1 strain, but then reduced Fe(III) faster than the parental strain (Fig. 1).

An inoculum from a DL-1/∆OmcS culture, taken after approximately 35 mmol/L of Fe(III) oxide had been reduced, was spread on an agar-solidified medium with fumarate as the electron acceptor. One of the isolated colonies that grew on the solidified medium was picked and transferred to liquid acetate-fumarate medium. It was designated strain JIB. An inoculum of strain JIB grown with fumarate as the electron acceptor began reducing Fe(III) oxide after a short lag period with a maximum rate of Fe(III) reduction that was substantially faster than the DL-1 wild-type strain (Fig. 1). Thus, deleting OmcS led to a strain that was more effective than the parental strain in Fe(III) oxide reduction.

**Fig 2:**
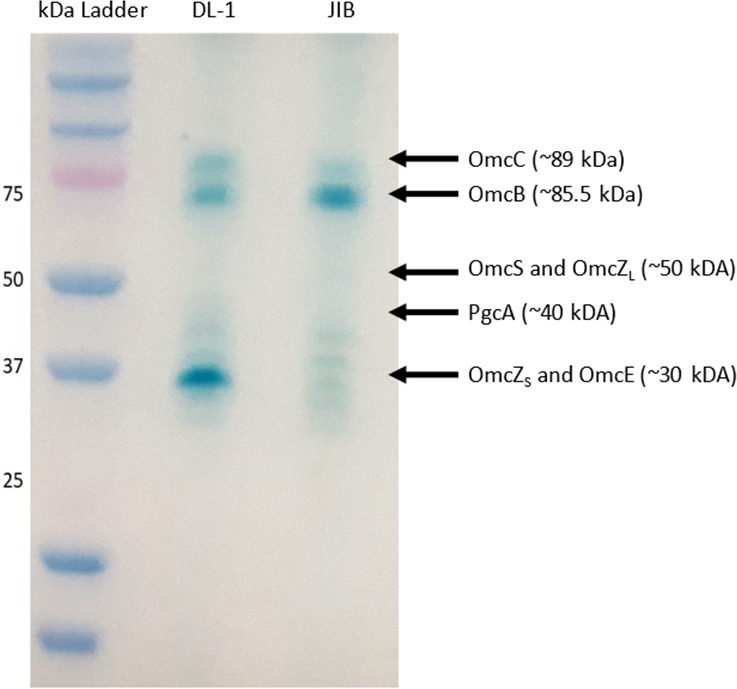
Heme-stain of SDS-PAGE separated outer-surface proteins of the DL-1 wild-type strain and strain JIB grown with Fe(III) oxide as the sole electron acceptor. Five micrograms of cell protein of each strain were processed for comparison. Designations on the right display predicted locations for *c*-type cytochromes involved in extracellular electron transfer.

**Fig 3:**
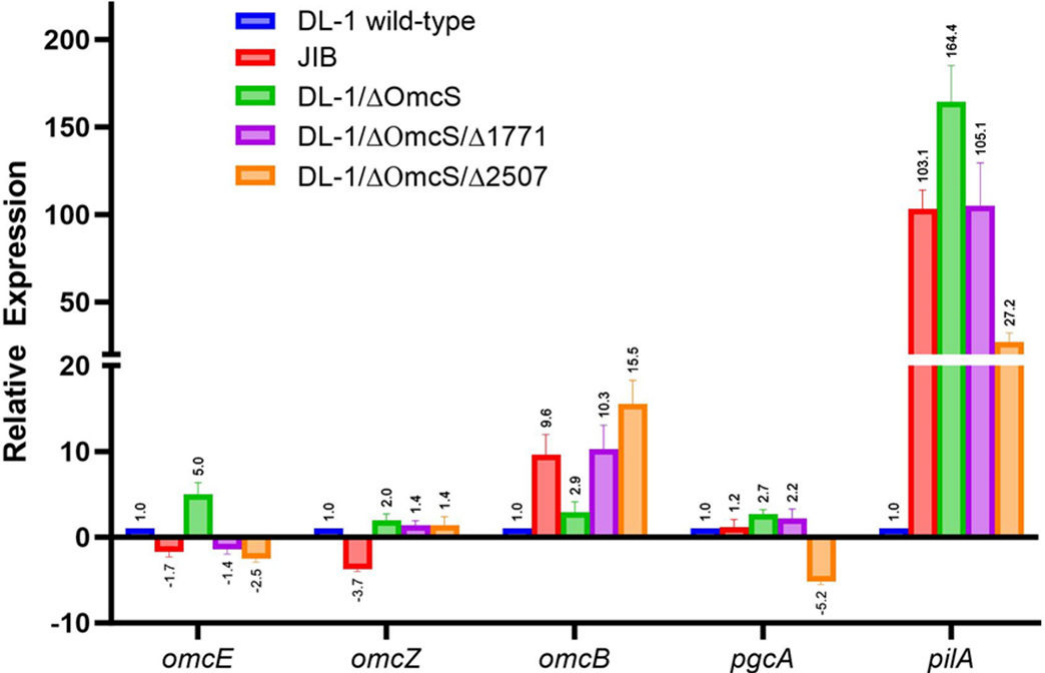
Relative transcript abundance of genes for multi-heme *c*-type cytochromes potentially involved in Fe(III) oxide reduction and the PilA gene. Relative expression ratios were determined with the 2^−ΔΔCt^ method, using the housekeeping gene *proC* for data normalization. Error bars show the standard deviation of the mean for technical triplicates and triplicate biological replicates.

Comparison of heme-stained outer surface proteins revealed that strain JIB did not compensate for the loss of OmcS with greater expression of the filament-forming outer-surface cytochromes OmcE and OmcZ (Fig. 2). Consistent with this observation, transcript abundance for the OmcE and OmcZ genes was lower in strain JIB than strain DL-1 (Fig. 3). In some instances, adaption for faster Fe(III) oxide reduction has been associated with increased expression of PgcA, a 50 kDa *c*-type cytochrome loosely associated with the outer membrane (37, 38). However, PgcA was not detected in strain JIB (Fig. 2), consistent with low *pgcA* transcript abundance in this strain (Fig. 3). Heme-staining indicated that the *c*-type cytochrome OmcB was more abundant in strain JIB than strain DL-1 (Fig. 2), consistent with a 9.6-fold higher *omcB* gene transcript abundance in strain JIB (Fig. 3). OmcB is partially embedded in the outer membrane and exposed on the outer surface as a component of a porin-cytochrome complex essential for electron transport across the outer membrane (39–42).

**Fig 4:**
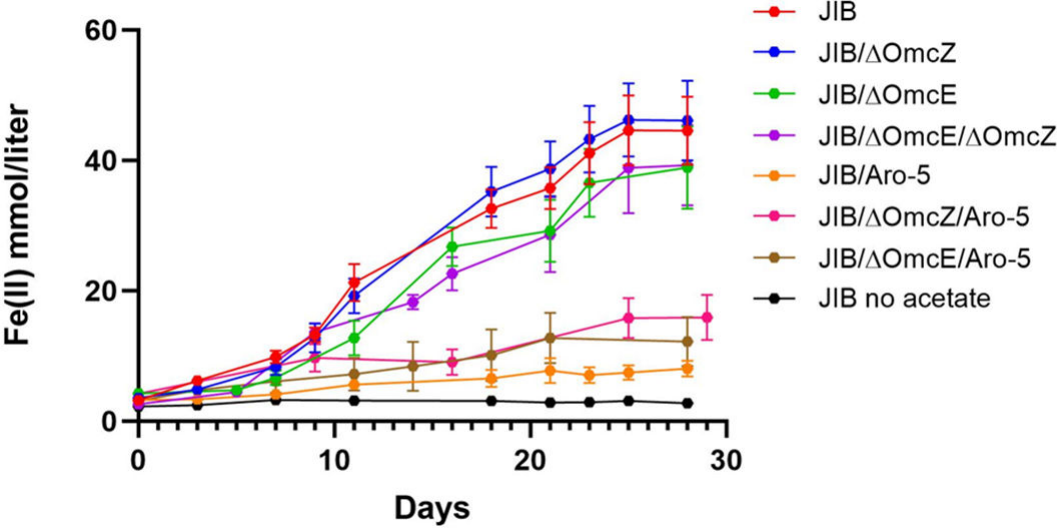
Fe(II) production from Fe(III) oxide reduction over time in mutant strains. Data are the means and standard deviation of triplicate cultures of each strain.

Deleting the genes for OmcZ or OmcE in strain JIB, either individually or within the same strain, did not inhibit Fe(III) oxide reduction (Fig. 4). These results further demonstrate that the JIB strain did not adapt to reduce Fe(III) oxide by increasing expression of OmcZ or OmcE filaments and that OmcZ and OmcE do not play major roles in extracellular electron transfer to Fe(III) oxide. These findings are consistent with previous results in which deleting these genes from strain DL-1 did not inhibit Fe(III) oxide reduction (12, 13, 43). The lack of appearance of other *c*-type cytochromes in the outer surface protein fraction (Fig. 2) also indicated that other cytochrome filaments were not substituting for the loss of OmcS.

### Aromatic-rich PilA required for Fe(III) oxide reduction

**Fig 5:**
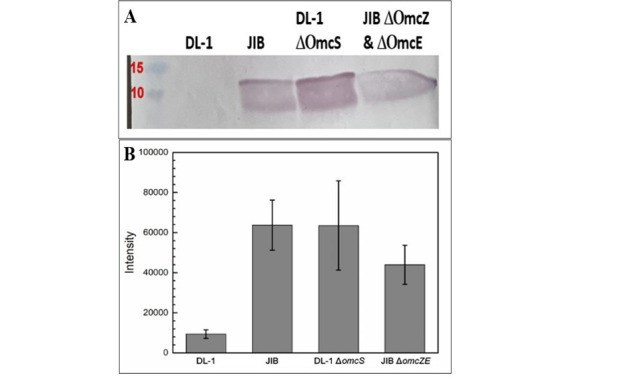
PilA abundance in outer cell surface protein preparations determined with a PilA-specific antibody. (**A**) Western blot analysis; (**B**) intensities from dot blot analysis calculated with ImageJ. Results are the means and standard deviation for dot blot analyses on preparations from three cultures of each strain.

PilA, the pilin protein proposed to assemble into e-pili (15, 22, 24, 25, 27), was more highly expressed in strain JIB and strain DL-1/∆OmcS than in the parental DL-1 strain (Fig. 5). Compared to strain DL-1, the PilA gene transcript abundance and PilA in outer-surface protein preparations (Fig. 3) were more than 100-fold and threefold higher, respectively, in both strains JIB and DL-1/∆OmcS.

It has previously been demonstrated that replacing the native PilA gene with an “Aro-5” pilin gene yields strains with poorly conductive 3 nm pili (15, 20, 28). Strains JIB/Aro-5, JIB/∆OmcE/Aro-5, and JIB/∆OmcZ/Aro-5 were constructed. Relatively little Fe(II) accumulated in Fe(III) oxide cultures of the strains expressing the Aro-5 pilin, indicating that the capacity for Fe(III) oxide reduction was severely impacted (Fig. 4). This result is consistent with previous studies that have demonstrated that *G. sulfurreducens* strains expressing genes encoding poorly conductive pili are deficient in long-range extracellular electron transfer (15, 16, 20, 21, 36).

**Fig 6:**
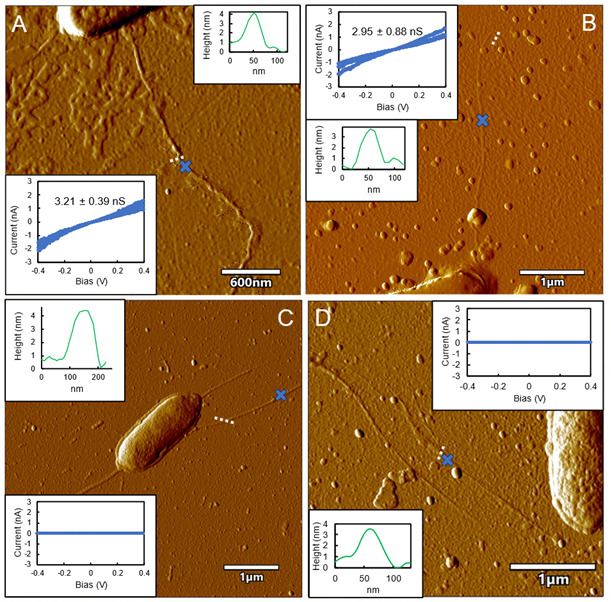
Atomic force microscopy of filaments emanating from cells demonstrating the expression of conductive filaments in a *G. sulfurreducens* strain in which genes for all three known filament-forming cytochromes were deleted, as well as the expression of poorly conductive filaments when an aromatic-poor pilin was expressed. All filaments had a diameter of ca. 3 nm. (**A and B**) Strain JIB/ΔOmcZ/ΔOmcE, which expresses the native pilin gene. (**C**) Strain JIB/ΔOmcZ/Aro-5, which is modified with the “Aro-5” gene that encodes an aromatic-poor pilin. (**D**) Strain JIB/ΔOmcE/Aro-5, which is modified with the “Aro-5” gene that encodes an aromatic-poor pilin. White lines designate where the diameter (height) measurements were made. Blue Xs designate the point where conductance measurements were made. Conductance data are the mean and standard deviation of triplicate measurements. Images are representative of measurements on filaments of at least nine cells of each strain.

Filaments emanating from cells were evaluated in more detail with strain JIB/∆OmcE/∆OmcZ to avoid the possibility that any of the filaments observed were the cytochrome filaments that have been proposed to be involved in extracellular electron transfer. The filaments detected with atomic force microscopy had a height of 3 nm (Fig. 6) with a conductance of ca. 3 nS, consistent with the 3 nm diameter and conductance of PilA filaments expressed in *E. coli* (22), and the conductance of 3 nm filaments emanating from *G. sulfurreducens* expressing wild-type PilA (15). Both strains JIB/∆OmcE/Aro-5 and JIB/∆OmcZ/Aro5 also expressed 3 nm diameter filaments, but the filament conductance was greatly diminished and difficult to accurately quantify (Fig. 6), consistent with the expectation that expression of an aromatic-poor pilin yields pili with low conductance (15, 16, 20, 21, 36). The correspondence between the expression of poorly conductive 3 nm diameter filaments and the nearly complete inhibition of Fe(III) oxide reduction suggests that 3 nm diameter filament conductivity is essential for effective long-range extracellular electron transport.

### Accelerated Fe(III) oxide reduction associated with mutations increasing expression of OmcB

The difference in the short lag period prior to the initiation of Fe(III) oxide reduction in strain JIB versus the long lag in strain DL-1/∆OmcS suggested that genomic mutations may have accumulated to facilitate growth on Fe(III) oxide. Whole-genome sequencing of strain JIB revealed mutations in three genes predicted to result in amino acid changes to proteins encoded in the strain DL-1 genome. Sanger sequencing confirmed that the mutations were not present in the unadapted DL-1ΔOmcS strain (Table 1). One of the mutations in strain JIB was in GSU3378, which is predicted to code for a glutamate-ammonia ligase adenylyltransferase. This protein is not expected to play a role in extracellular electron transfer, and a previous study has speculated that this gene may be inactive in *G. sulfurreducens* (44). Thus, this mutation was not investigated further.

**TABLE 1:** Mutations found in the JIB strain of *G. sulfurreducens* compared with the DL-1/ΔOmcS strain verified by Sanger sequencing

Mutated gene	Annotation	JIB mutation
GSU1771	DNA/RNA binding protein	IS element; interruption of GSU1771 after 94 bp by Transposase ISGsu4 (1,275 bp insertion)
GSU2507	Sensor histidine kinase	Deletion of a G at 1,356 bp GTG → GTx Results in a frameshift and early stop codon after 416 amino acids (wild-type protein has 621 amino acids)
GSU3378	Glutamate-ammonia ligase adenylyltransferase	Deletion of 27 bp after 2,484 bp Results in in-frame deletion Loss of amino acids EGYVYKLDT after 827 amino acids

Another mutation was in GSU1771 (Table 1), which codes for a *Streptomyces* antibiotic regulatory protein family transcriptional regulator, known to impact the expression of multiple cytochromes and PilA in strain DL-1 (45). Deletion of GSU1771 from strain DL-1/∆OmcS generated strain DL-1/∆OmcS/∆1771. OmcB gene transcript abundance in strain DL-1/∆OmcS/∆1771 was 10-fold higher than in strain DL-1/∆OmcS (Fig. 3). Transcripts were not significantly higher for OmcE, OmcZ, or PgcA genes in strain DL-1/∆OmcS/∆1771 and PilA gene transcripts were lower than in strain DL-1/∆OmcS (Fig. 3).

The other mutation in strain JIB was in GSU2507 (Table 1), which encodes a sensor histidine kinase. Deletion of GSU2507 in the DL-1/ΔOmcS strain generated strain DL-1/∆OmcS/∆2507. Like strain DL-1/∆OmcS/∆1771, strain DL-1/∆OmcS/∆2507 had increased OmcB gene transcript abundance, without increased expression of the other cytochrome genes evaluated, and a decrease in PilA gene transcripts (Fig. 3). These results suggest that the GSU1771 and GSU2507 mutations found in strain JIB may have contributed to the high levels of OmcB in this strain (Fig. 2).

**Fig 7:**
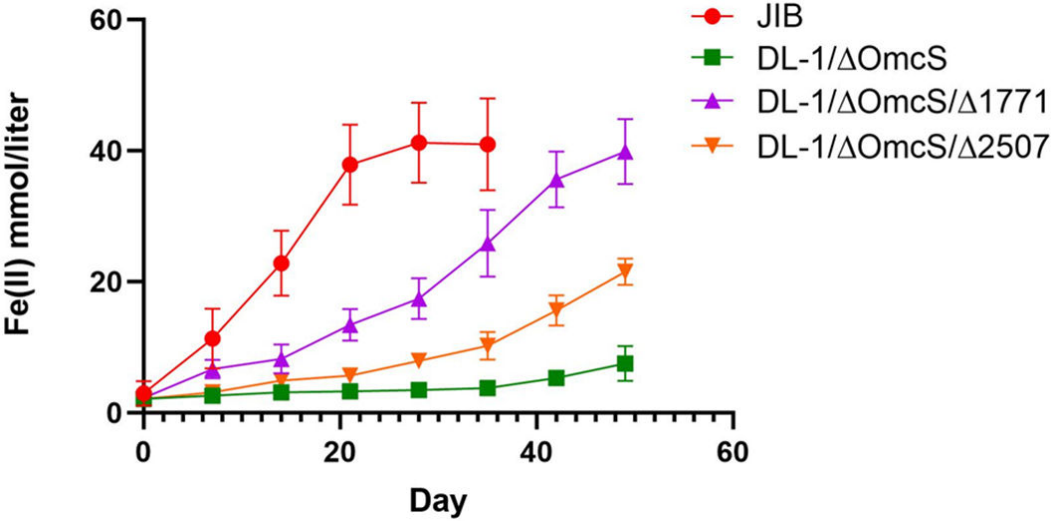
Introducing mutations found in strain JIB into strain DL-1/∆OmcS reduces the lag period for the initiation of Fe(II) production from Fe(III) oxide reduction. The results are the means and standard deviation of triplicate cultures for each strain.

OmcB is an essential component of the primary porin cytochrome conduit for electron transfer across the *G. sulfurreducens* outer membrane, and a likely electron donor to conduits for long-range electron transfer to electron acceptors at a distance from the cell (18, 46). Like strain JIB, both strain DL-1/∆OmcS/∆1771 and strain DL-1/∆OmcS/∆2507 did not exhibit a substantial lag period before initiating Fe(III) oxide reduction (Fig. 7). The inocula for the Fe(III) oxide reduction studies were grown with fumarate as the electron acceptor and the gene for OmcB is not highly expressed during growth on fumarate compared to growth on Fe(III) (47). Thus, the mutations leading to higher OmcB expression during growth on fumarate may have poised strain JIB to reduce Fe(III) oxide without a significant lag period.

## DISCUSSION

The results demonstrate that there is no rigorous functional evidence that supports the concept that cytochrome filaments are the primary conduits for long-range electron transfer in microbes. Genetic modifications that prevent the expression of cytochrome filaments had no negative impact on Fe(III) oxide reduction. In contrast, expressing pili with poor conductance in strains in which filament-forming cytochrome genes had been deleted inhibited Fe(III) oxide reduction. This result is consistent with the concept that e-pili are the key conduits for long-range electron transfer.

As detailed in the Introduction, multiple lines of evidence have already ruled out a primary role for cytochrome filaments in long-range electron transport through *G. sulfurreducens* biofilms. The only experimental evidence for cytochrome filament long-range electron transfer has been the finding (12) that OmcS is required for *G. sulfurreducens* Fe(III) oxide reduction. However, we found that the same OmcS-deficient strain previously reported to be unable to reduce Fe(III) oxide (12) not only readily reduced Fe(III) oxide, but upon adaptation, reduced Fe(III) oxide faster than the parental strain. This is analogous to an OmcS gene deletion having no impact on current production (13) and increasing *G. sulfurreducens* biofilm conductivity (11). We found that deleting the genes for OmcE and OmcZ, the other *G. sulfurreducens* filament-forming cytochromes, along with OmcS, also had no impact on Fe(III) oxide reduction. These results indicate that cytochrome filaments are not the primary conduits for long-range extracellular electron transport to Fe(III) oxides.

The results also further demonstrate that e-pili are comprised of PilA. The 3 nm diameter conductive filaments had the same morphology and conductance as the filaments produced when *G sulfurreducens* PilA is expressed in *E. coli*. Furthermore, the conductance of the 3 nm diameter filaments dropped substantially when the aromatic amino acid content of PilA was decreased. These results are consistent with multiple lines of additional evidence, detailed in the Introduction, that PilA assembles into e-pili. The results are inconsistent with the hypothesis that the 3 nm diameter filaments are comprised of extracellular DNA or another as-yet-to-be-described cytochrome that also forms filaments.

The energetic cost associated with the biosynthesis of cytochrome filaments suggests that they serve an important function. The selection for mutations that increased expression of OmcB in the OmcS-deficient mutant suggests that OmcS, which has been observed associated with the cell surface (48) as well as forming filaments emanating from cells (15), facilitates electron transfer at the outer cell surface, possibly enhancing electron transfer from OmcB to e-pili. If so, then the greater abundance of PilA in outer-surface protein preparations of strains not expressing OmcS could also reflect the need to compensate for this lost role of OmcS.

*G. sulfurreducens* cytochromes can serve as capacitors to store electrons to enable continued respiration and ATP generation when cells are not in contact with a sufficient quantity of extracellular electron acceptor (49). An extracellular location for this capacitance, such as that afforded with OmcS filaments, could overcome space limitations for establishing capacitance within cells.

However, without further experimental functional data, detailed speculation on the role of cytochrome filaments seems unwarranted. Filaments with the unique morphology of OmcS filaments accounted for only ca. 10% of the filaments emanating from wild-type *G. sulfurreducens* cells (15). This relatively low level of filament expression may further increase the difficulty in functional experimental design. Furthermore, *G. sulfurreducens* not only expresses multiple cytochrome filaments but also a diversity of other outer-surface redox-active proteins, that may provide additional extracellular electron transfer routes and possibly further complicate the elucidation of cytochrome filament function. The development of experimental approaches that can directly measure physiological electron transport along cytochrome filaments emanating from cells would greatly facilitate functional analysis.

A diversity of microbes are likely to produce cytochrome filaments (6). Studies of cytochrome filament function might be simpler in an alternative native host with a smaller repertoire of outer-surface redox proteins than *G. sulfurreducens*. Another approach might be a heterologous expression of filament-forming cytochromes in an easily grown, genetically tractable host that does not have cytochrome filament genes. Such strains may also be suitable for producing cytochrome filaments as a sustainable material for electronic device applications.

## MATERIALS AND METHODS

### Cell culture and growth conditions

*G. sulfurreducens* DL-1 (ATCC 51573), *G. sulfurreducens* DL-1 Δ*omcS::spec* (12), *G. sulfurreducens* DL-1 Aro-5::*gent* (20), *G. sulfurreducens*Δ*omcZ::kan* (13), and *G. sulfurreducens*Δ*omcE::kan* (12) were obtained from the Lovley lab culture collection. All cultures were incubated under anaerobic conditions (N_2_:CO_2_, 80:20, vol/vol) at 30°C with 10 mM acetate provided as the electron donor and fumarate (40 mM) or Fe(III) oxide (80 mmol/L) as the electron acceptor. For mutant selection, cells were plated onto acetate-fumarate agar containing the appropriate antibiotic in an anaerobic chamber under N_2_–CO_2_–H_2_ (83%:10:7%). Fe(III) oxide reduction was quantified by acidifying samples to dissolve minerals and determining Fe(II) with a ferrozine assay (50).

### JIB genome sequencing

Sequencing and screening for polymorphisms of strain JIB were performed at MR DNA Lab (Shallowater, TX, USA, www.mrdnalab.com). Genomic DNA was extracted, and libraries were prepared with the Nextera DNA Flex library preparation kit (Illumina) following the manufacturer’s instructions. The initial concentration of DNA was evaluated with the Qubit dsDNA HS Assay Kit (Life Technologies). In order to remove small fragments, the DNA samples were treated with the DNeasy PowerClean Pro Cleanup Kit (Qiagen, Valencia, CA), and concentrations were again evaluated using the Qubit dsDNA HS Assay Kit. Fifty nanograms of DNA were used to prepare the libraries. The samples underwent simultaneous fragmentation and the addition of adapter sequences. These adapters were utilized during a limited-cycle PCR in which unique indices were added to the sample. Following the library preparation, the final concentration of the libraries was measured using the Qubit dsDNA HS Assay Kit, and the average library size was determined using the Agilent 2100 Bioanalyzer (Agilent Technologies). The libraries were then pooled in equimolar ratios of 0.6 nM, and sequenced paired-end for 500 cycles using the NovaSeq 6000 system (Illumina). Polymorphisms were screened for using the *G. sulfurreducens* PCA (AEO17180) (51) reference sequence. Potential mutations were amplified with PCR from both JIB and DL-1/ΔOmcS and verified with Sanger sequencing.

### Mutant strain construction

All primers used for mutant construction are listed in Table 2. To construct the JIB/ΔOmcZ mutant strains, the primer pair OmcZ_fwd and OmcZ_rev were used to amplify the mutated region, including the kanamycin resistance cassette, from the previously described DL-1/ΔOmcZ strain (13). For the construction of the JIB/ΔOmcE mutant, the primer pair OmcE_fwd and OmcE_rev were used to amplify the mutated region, including the kanamycin resistance cassette, from the previously described DL-1/ΔOmcE strain (12). For the construction of JIB/Aro-5, JIB/ΔOmcZ/Aro-5, and JIB/ΔOmcE/Aro-5, the Aro-5-*pilA* gene, including the gentamycin resistance cassette, were amplified from the DL-1/Aro-5 strain (20). Following PCR amplification, products were purified using the QIAquick gel extraction kit (Qiagen) and verified by Sanger sequencing.

**TABLE 2:** Primers used for mutant construction and quantitative PCR^*a*^

Primer name	Purpose	Sequence (5′ to 3′)
OmcZ_fwd	JIB Δ*omcZ::kn^R^* JIB Aro-5::*gent^R^*Δ*omcZ::kn^R^* JIB Δ*omcZ::kn^R^* Δ*omcE::gent^R^*	GCGTCCTGCTGCGGTTGTCCG
OmcZ_rev		CTTGACGCGACCGCCGTGCCG
OmcE_fwd	JIB Δ*omcE::kn^R^* JIB Aro-5::*gent^R^*Δ*omcE::kn^R^*	GAGCCTGTCGGACTTGGAAAT
OmcE_rev		TTGTAGTTGGTCTGGGGGTCG
Aro-5_fwd	JIB Aro-5::*gent^R^* JIB Aro-5::*gent^R^*Δ*omcZ::kn^R^* JIB Aro-5::*gent^R^*Δ*omcE::kn^R^*	CAGGAAGCTTACGTTCCGTTCTTTCC
Aro-5_rev		CAGATGACTACTGCGACTTCCACTCG
GSU1771_up_fwd	*1771*::*gent^R^*	GACCCAGAGTTTCGGGAAAGGC
GSU1771_up_rev	*1771*::*gent^R^*	TATCCTAGGCATCGCGATACACCCTTACCT
GSU1771_dn_fwd	*1771*::*gent^R^*	TATCCTAGGGCGGGGATGGCTCATC
GSU1771_dn_rev	*1771*::*gent^R^*	TACATTGGACAACCCTGTGGC
GSU2507_up_fwd	*2507::gent^R^*	AAGTCCAGCAGGTAGGATGC
GSU2507_up_rev	*2507::gent^R^*	CTACCTAGGCATGGACAAAGCGGGACTCG
GSU2507_dn_fwd	*2507::gent^R^*	CTACCTAGGACGAGTATGGCCAGAAGGGT
GSU2507_dn_rev	*2507::gent^R^*	GTAGCGCCTGCCGATGGATT
mcE_up_fwd	JIB Δ*omcZ::kn^R^* Δ*omcE::gent^R^*	TGTCGACTTGTGCAGGTTAATGGT
mcE_up_rev		TATCCTAGG CATTCTGCGTTCTCCTTTCAC
mcE_dn_fwd		TATCCTAGGTCTGGGTTGCCACAAGAAGTAG
mcE_dn_rev		CTTAGTATCAAAGGAGCCCCACTCG
proC_f	qRT-PCR	GGAATACCTGCACGGCACCT
proC_r	qRT-PCR	GCAACCATGCCACGGAACAC
pilA_f	qRT-PCR	TCGTCGTTGCGATCATCGGT
pilA_r	qRT-PCR	TGCGGACTCAAGAGCAGTCT
mcE_q_f	qRT-PCR	CGCCACATTCAAGGCGACGAAC
mcE_q_r	qRT-PCR	TAAAGCCCTGCTTGACCGGCAC
mcZ_q_f	qRT-PCR	TCCGAGTACGACGCCGGTAT
mcZ_q_r	qRT-PCR	GGAGAGCGGACCGTTGATGG
mcB_f	qRT-PCR	GTGCCGTTACCGCCATTACCAG
mcB_r	qRT-PCR	AGCTCCCCAGGATCGCCAATAC
pgcA_f	qRT-PCR	GGCAGTGGCTCCTACTCCTA
pgcA_r	qRT-PCR	GTCTGGATGTCGGAATTCGT

^*a*^ The AvrII restriction site is underlined. qRT-PCR, quantitative real-time polymerase chain reaction.

For the construction of JIB/ΔOmcZ/OmcE, DL-1/ΔOmcS/Δ1771, and DL-1/ΔOmcS/Δ2506, OmcE, GSU1771, or GSU2506 were replaced with a gentamycin resistance gene as previously described (52). Primer pairs were designed flanking ~500 bp of the upstream and downstream regions of the target genes. PCRs were performed using Phusion High-Fidelity DNA Polymerase (NEB, Beverly, MA), and PCR products were digested with the *AvrII* (CCTAGG) (NEB) restriction endonuclease. The digested products were purified and ligated using T4 DNA ligase (NEB). The 1,000 bp ligation product was cloned into the pCR2.1 TOPO cloning vector and cloned sequences were verified using Sanger sequencing. The recombinant plasmid was then digested with *AvrII*, and a gentamycin resistance cassette was ligated into the plasmids. Plasmids were linearized and ethanol precipitated to a final concentration of at least 1,000 ng/µL.

Electrocompetent cells were prepared as previously described (52) and the purified PCR products or linearized plasmid DNA were electroporated into the appropriate competent cells at 14.7 kV/cm for 6 ms (52). Cells were recovered for 8 h in the fumarate-acetate medium at 30°C and then plated on fumarate-acetate agar containing the appropriate antibiotic for selection in an anaerobic chamber. Mutations were confirmed using PCR and Sanger sequencing.

### Real-time quantitative polymerase chain reaction

Triplicate cultures of DL-1 wild-type, strain JIB, DL-1/ΔOmcS, DL-1/ΔOmcS/Δ1771, and DL-1/ΔOmcS/Δ2507 were grown in fumarate-acetate medium and cells were pelleted by centrifugation at 3,000 × *g* for 20 min at 4°C during late exponential phase when the OD (600 nm) reached approximately 0.4. RNA was extracted using the RNeasy Mini Kit (Qiagen) and treated with DNase (Ambion, Austin, TX). PCR was used to verify that the RNA was free of DNA contamination. RNA was quantified using a NanoDrop ND-1000 spectrophotometer (NanoDrop Technologies, Wilmington, DE). Complementary DNA was generated from mRNA using the Invitrogen SuperScript IV First Strand Synthesis System (ThermoFisher Sci).

All primers used for quantitative real-time polymerase chain reaction (qRT-PCR) are shown in Table 2. The constitutively expressed housekeeping gene *proC*, which codes for pyrroline-5-carboxylate reductase, was used as an external control (53). Power SYBR green PCR master mix (Applied Biosystems, Foster City, CA) and an ABI 7500 real-time PCR system were used to amplify and quantify all PCR products. Each reaction mixture consisted of forward and reverse primers at a final concentration of 200 nM, 5 ng of gDNA, and 12.5 µL of Power SYBR green PCR master mix (Applied Biosystems). Relative levels of expression of the studied genes were calculated by the 2^−ΔΔCT^ threshold cycle (CT) method (54).

### Outer cell surface SDS-PAGE, heme staining, and PilA

*G. sulfurreducens* DL-1 and JIB strains were cultured in 100 mL bottles containing Fe(III) oxide-acetate medium and outer cell surface proteins were collected when Fe(II) concentrations reached approximately 30 mmol/L. Outer surface proteins were collected as previously described (12, 55) and protein concentrations were measured with a bicinchoninic acid assay. Proteins were separated on a glycine-buffered 12.5% polyacrylamide gel and stained for heme as previously described (56).

To determine the presence of PilA in outer surface proteins, cells were cultured in acetate-fumarate medium to an OD of 0.4, outer surface proteins were recovered as described above, and 20 µL of the extracted protein were mixed with 20 µL 2× Laemmli loading buffer containing 5% β-mercaptoethanol, which was heated at 95°C for 10 min. The samples were loaded onto a 4%–20% Mini-Protean TGX precast gel (Bio-Rad), then blotted onto a nitrocellulose membrane using a semidry transfer cell (Bio-Rad). The membrane was subsequently blocked using a 3% BSA solution and incubated with a previously described anti-PilA antibody (27). The membrane was then washed and exposed to horseradish peroxidase-conjugated goat anti-rabbit secondary antibodies (Invitrogen). The membrane was washed again, and the blots were developed at ambient temperature using a 1-Step Ultra TMB Blotting Solution substrate (Thermo Scientific, Rockford, IL, USA).

For dot blot analysis, protein samples (10 µL each) were applied to a nitrocellulose membrane and allowed to dry at room temperature. The membrane was then treated with a 3% BSA blocking solution for 30 min, followed by incubation with anti-PilA antibodies. The membrane was washed tris buffered saline with 0.5% Tween and exposed to the goat anti-rabbit secondary antibody. After another washing step, the blots were developed using 1-Step Ultra TMB Blotting Solution substrate (Thermo Scientific) at ambient temperature. ImageJ software was utilized to assess the difference in intensity for each blot (57).

### Atomic force microscopy

For examination with atomic force microscopy, 25–40 µL of the culture was drop-cast onto a conductive 35 nm platinum-coated silicon wafer as previously described (15). After 12 min, the excess liquid was wicked off with filter paper, the sample was rinsed with deionized water, and the excess liquid was removed with filter paper. The samples were equilibrated with the chamber humidity (ca. 40%) and visualized with a Cypher atomic force microscope (Asylum Research, Oxford Instruments) as previously described (15). Initially, the cells and filaments were viewed under tapping mode (AC-air topography) with a Pt/Ir-coated tip (PtSi-FM, NanoWorld) at approximately 2.0 N/m spring force constant and 70 kHz resonance frequency. After filament height determination, contact mode was engaged with the tip gently placed on top of the filament (force 30 ± 1.1 nN) as the translatable top electrode. Quadruplicate amplitude voltage sweeping of ±0.4 V at a frequency of 0.99 Hz was applied and averaged for I–V response curves. The conductance of the filament was calculated using the linear slope between 0.2 V and −0.2 V in the I–V response.

## Data Availability

The JIB genome sequence reads have been submitted to the SRA NCBI database under BioProject PRJNA1010368 and Biosample SAMN37185955.
